# Wallenberg’s syndrome and symptomatic trigeminal neuralgia

**DOI:** 10.1007/s10194-011-0305-9

**Published:** 2011-02-10

**Authors:** Carlos M. Ordás, María L. Cuadrado, Patricia Simal, Raúl Barahona, Javier Casas, Jordi Matías-Guiu Antem, Jesús Porta-Etessam

**Affiliations:** Servicio de Neurología, Hospital Clínico San Carlos, Universidad Complutense, Profesor Martín Lagos s/n, 28040 Madrid, Spain

**Keywords:** Trigeminal neuralgia, Facial pain, Wallenberg’s syndrome, Lateral medullary infarction, Brainstem infarction

## Abstract

Symptomatic trigeminal neuralgia due to a brainstem infarction is said to be rare. However, facial pain is not uncommon in Wallenberg’s syndrome. Facial pain related to a Wallenberg’s syndrome may be either persistent of intermittent, and occasionally occurs in brief attacks. Here, we report a patient with a right lateral medullary infarction who started having first division trigeminal neuralgia 1 month after the stroke. The pain paroxysms were suppressed with gabapentin.

## Introduction

Symptomatic trigeminal neuralgia (TN) is caused by a demonstrable structural lesion other than vascular compression, typically posterior fossa tumors or multiple sclerosis [1, 2]. TN due to a brainstem infarction is considered to be rare. Yet, Wallenberg’s syndrome is commonly associated with ipsilateral facial pain [3]. This pain may be either persistent or intermittent, sometimes occurring in brief attacks that may reproduce TN features. Here, we report the case of a patient with Wallenberg’s syndrome who started having shock-like painful paroxysms in the first division of the trigeminal nerve (V1).

## Case report

A 41-year-old man visited our hospital reporting a 48-h lasting pain localized on the right side of his neck. Three hours before entering the emergency room, the patient felt sudden dizziness with gait instability and clumsiness of his right limbs. His past medical history was remarkable for non-controlled hypertension as well as frequent alcohol and occasional cocaine intake. The patient denied any drug intake in the past few days.

**Fig. 1:**
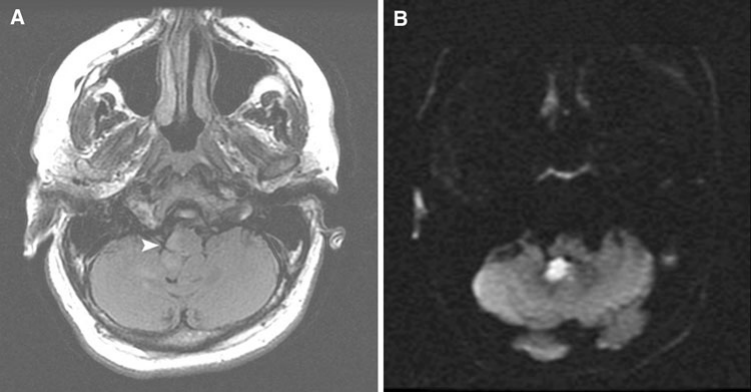
Magnetic resonance imaging (MRI). **a** Axial T2-weighted FLAIR MRI shows slight hyperintensities in the right lateral medulla and right cerebellum. **b** Diffusion-weighted MRI demonstrates restricted water motion in the lesion shown in **a**, indicating recent infarction

**Fig. 2:**
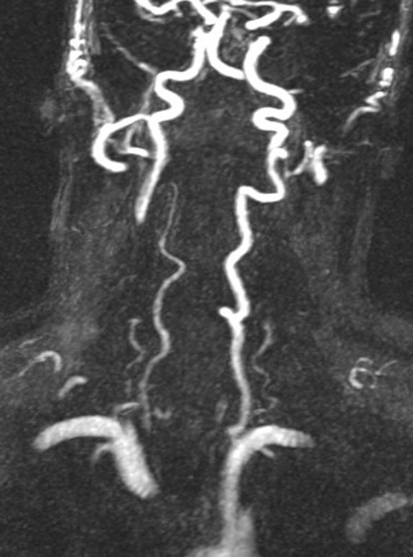
Magnetic resonance angiography (MRA). Coronal maximum intensity projection from MRA reveals occlusion of the right vertebral artery, which is likely due to artery dissection

On a general physical examination, the only relevant finding was a blood pressure measurement of 205/120 mmHg. The neurologic examination revealed moderate dysarthria, a right Horner syndrome, vertical nystagmus in all extreme gaze positions, an absent right gag reflex, right facial and left two limb hypoalgesia and thermoanesthesia, mild paresis of the right limbs, and severe right limb ataxia. An early brain computed tomography (CT) scan did not detect any significant abnormalities, but magnetic resonance imaging (MRI) showed a recent infarction in the right lateral medulla (Fig. 1a, b). In addition, complete occlusion of the right vertebral artery was demonstrated on magnetic resonance angiography (MRA; Fig. 2). This occlusion was attributed to spontaneous artery dissection. There was no evidence of a vascular contact at the root entry zone of the right trigeminal nerve.

Forty-eight hours after admission, the patient suffered from aspiration pneumonia with breathing compromise and required intubation at the Intensive Care Unit (ICU). After several lung complications, he was eventually extubated and returned to our hospital ward 1 month after the stroke. At this moment we found the same neurologic signs he presented at onset, though milder, including some sensory impairment on the right side of his face. Two days after leaving the ICU, our patient started having brief electric shock-like pains in the whole distribution of the first division (V1) of the right trigeminal nerve. Pain attacks fulfilled diagnostic criteria for symptomatic TN, according to the International Classification of Headache Disorders, 2nd edition (ICHD-II) [1]. They lasted for 3–5 s, occurred at least 10 times per hour, and were given a score of 8 out of 10 on a visual analogue pain intensity scale. No autonomic symptoms or trigger points were noticed. Between the painful paroxysms, the patient was free of pain. Treatment with gabapentin was initiated. While being on a dose of 300 mg t.i.d., the patient experienced a decrease in both pain frequency and intensity plus a restriction of the pain to a more circumscribed periocular area. When gabapentin was titrated up to 600 mg t.i.d, the pain was finally controlled. At discharge he maintained this dose and had no recurrences of the pain through a 2-month follow-up.

## Discussion

It is widely accepted that the commonest cause of TN is compression of the trigeminal root entry zone by a blood vessel. The term “classical TN” is applied to those cases with potential vascular compression or unknown etiology. When a causative lesion other than vascular compression is demonstrated, a diagnosis of “symptomatic TN” is made [1]. Overall, symptomatic TN accounts for 15% of cases of NT, and the majority of these are caused by cerebellopontine angle tumors or multiple sclerosis [2]. TN due to a brainstem infarction is said to be rare.

Although a brainstem infarction had been previously suggested as a possible cause of TN [4], it was Balestrino and Leandri who reported the first well-documented case in 1997. These authors discovered a small ischemic lacune at the right lateral part of the pons in a patient with a 4-year history of ipsilateral second division (V2) TN and slight sensory loss [5]. In 1998 Golby et al. described a patient who started having lancinating pain in V1 and V2 distributions on the left side. He had experienced sudden hemifacial numbness 1 year before, and MRI demonstrated an old ischemic lesion at the root entry zone of the trigeminal nerve [6]. In 2004 Peker et al. reported a female patient who suddenly developed a neuralgic pain in her left chin and cheek. A slight hypoesthesia was found in V2 and V3 territories, and MRI showed a chronic infarction transecting the central trigeminal pathways within the pons [7]. In 2006, Warren et al. described a female patient who presented with concurrent left trigeminal and glossopharyngeal neuralgia. Delayed MRI disclosed a left lateral medullary infarction [8]. Katsuno and Teramoto have recently reported a patient with sudden onset of facial numbness and TN in the right V2 dermatome. MRI showed an acute pontine infarction, just at the root entry zone of the right trigeminal nerve [9]. The pathogenesis of TN secondary to a brainstem ischemic lesion is uncertain. The main hypothesis states that demyelination in the central trigeminal pathways would cause ephaptic transmission and thereby abnormal electric impulses, as it happens in multiple sclerosis. Other theories postulate that irritation of trigeminal structures in a bed made from the scar would be the base of excessive reactivity in an epileptic-like manner.

Facial pain may be a feature of Wallenberg’s lateral medullary syndrome, along with a decrease of pain and temperature sensation of the face. Other clinical features include vertigo, eye movement disorders, an ipsilateral Horner’s syndrome, ipsilateral limb ataxia, and contralateral sensory deficit. In reviewing the literature, pain incidence rates following a lateral medullary infarction vary largely, with higher rates in follow-up observations. The pain may start just at the stroke onset, but most patients have a latency between 2 weeks and 6 months. Some patients feel a constant, boring, pain, while others describe short pain attacks that occur either spontaneously or related to an innocuous stimulus [3]. Therefore, Wallenberg syndrome is a typical cause of central post-stroke pain, and this pain may occasionally take the attributes of a symptomatic TN.

Among 12 patients with Wallenberg’s syndrome, Fitzek et al. found 6 patients with facial pain. The latency between the stroke and pain onset ranged between 1 month and 2 years. Facial pain was always ipsilateral to the infarction, and was mostly localized in the periorbital region. These patients reported short pain attacks lasting seconds to minutes, and three of them also had persistent pain. Five of them mentioned trigger factors [10]. Although a diagnosis of TN was not made, the shortest pain attacks—i.e., those lasting just a few seconds—apparently had typical TN features [11].

Our patient also had neuralgiform pain attacks in association with a Wallenberg’s syndrome. The painful paroxysms were brief and intense, fulfilling ICHD-II diagnostic criteria for symptomatic TN [1]. They started with a latency of 1 month after the stroke onset, which was in line with Fitzek’s series [10]. While the hypoesthesia extended through the territory of the three trigeminal branches, the pain occurred in a V1 distribution. This was also in accordance with Fitzek’s cases, whose pain was mostly centered in the periorbital region [10]. In contrast, classical TN usually starts in V2 or V3, and only very rarely affects V1 [1, 11]. The somatotopic arrangement of trigeminal fibers may possibly account for these topographical differences between TN due to nerve root compression and TN due to a lateral medullary infarction.

Lesions of the dorso-lateral medulla involve both the trigeminal descending tract and the trigeminal spinal nucleus, and this may lead to sensory impairment and pain in the ipsilateral face. In Fitzek’s clinical series of Wallenberg’s syndrome, patients with facial pain had lesions covering the trigeminal spinal tract and nucleus at the lower medulla, as demonstrated by MRI. In addition, the R2 blink reflex component was abnormal only in patients with facial pain. Likewise, sensory thresholds in the ipsilateral face were specifically elevated in those patients presenting with facial pain [3, 10].

Current treatment options for TN include several medical and surgical therapies. Carbamazepine and oxcarbazepine are considered first line drugs for classical TN. However, treatment of symptomatic TN is still unsettled. The existing studies all deal with TN associated with multiple sclerosis and are small open-label trials. Three trials including a total of 19 patients with multiple sclerosis have reported an effect of gabapentin alone or associated with carbamazepine [2]. Gabapentin might be also effective in symptomatic TN due to a brainstem infarction. In fact, gabapentin, pregabalin or amitriptyline are currently recommended for first line treatment in central neuropathic pain [12]. Our patient seemed to respond to gabapentin. Indeed, he experienced significant improvement when the drug was initiated, and had complete relief with further titration.

In conclusion, Wallenberg’s syndrome may be associated with symptomatic TN. Ischemic lesions covering the trigeminal spinal tract and nucleus at the lower levels of the medulla seem to be involved in the pathogenesis of the pain. Gabapentin might be an effective drug for symptomatic TN related to a lateral medullary infarction.
